# Laser-Tunable Printed ZnO Nanoparticles for Paper-Based UV Sensors with Reduced Humidity Interference

**DOI:** 10.3390/nano11010080

**Published:** 2021-01-02

**Authors:** Georges Dubourg, Marko Radović, Borislav Vasić

**Affiliations:** 1 Center for Sensor Technologies, BioSense Institute, University of Novi Sad, Zorana Đinđića, 21101 Novi Sad, Serbia; marrad@biosense.rs; 2 Institute of Physics Belgrade, University of Belgrade, Pregrevica 118, 11080 Belgrade, Serbia; bvasic@ipb.ac.rs

**Keywords:** paper-based device, UV sensors, ZnO nanoparticles, humidity resistance

## Abstract

Development of paper-based sensors that do not suffer with humidity interference is desirable for practical environmental applications. In this work, a laser processing method was reported to effectively modulate the cross-sensitivity to humidity of ZnO-based UV (Ultraviolet) sensors printed on paper substrate. The results reveal that the laser induced zinc oxide (ZnO) surface morphology contributes to the super-hydrophobicity of the printed ZnO nanoparticles, reducing humidity interference while enhancing UV sensitivity. Herein, this conducted research highlights for the first time that laser processing is an attractive choice that reduces the cross-sensitivity to water vapor in the UV sensing response of ZnO-based devices printed on paper, paving the way to low-cost and sophisticated paper-based sensors.

## 1. Introduction

For a long time, cellulose fiber paper has been used as the main support for storing, displaying, transferring information and connecting people in the form of missives, flyers or books. In recent years, however, its function as a writing medium has been declining with the evolution of information and communication technologies. This evolution drastically changed how people work, communicate and learn, gradually replacing the paper substrate with electronic support, such as computer, TV, e-book and e-library. Nevertheless, this has generated a considerable amount of electronic waste resulting in severe environmental issues. In order to address the environmental concerns and end-of-life disposal challenges, paper was reinvented as a building component in flexible electronics because it is disposable, recyclable, inexpensive and one of the most abundant organic materials. Therefore, paper-based substrate has been explored with the aim of developing a variety of components, such as electronic and optoelectronic devices [1,2], electrochemical biosensors [3,4,5] and physical sensors [6,7]. Among them, physical sensors which transduce physical parameters, such as mechanical and optical signals, into processable electrical signals represent a major category of paper-based devices and might play an important role in the upcoming era of wearable electronics.

Various functional nanomaterials have been employed in order to enhance the paper-based sensor performance. Among them, the zinc oxide (ZnO) nanostructures are a popular choice due to their multiple sensing modalities [8,9,10]. For instance, its direct wide bandgap and 60 meV high exciton energy make ZnO nanostructures the ideal materials for UV sensing applications [11,12]. Due to the growing importance of detecting/protecting UV light application in various fields, various reports have been published on the fabrication of low-cost ZnO-based UV sensors on paper substrate [13,14,15,16]. Although many of the reported paper-based UV sensors work very well in laboratory scale proof of principle experiments, they are still limited by their cross-sensitivity to background conditions, such as relative humidity than can vary in outdoor conditions, impeding their practical use in applications such as environmental monitoring. Indeed, it has been demonstrated that water vapor, present in the real environment for monitoring, strongly interacts with the surface or interface of a ZnO nanostructure, which leads to a significant fluctuation of its electrical and UV sensing properties [17,18,19]. Although the effect of humidity on UV sensing properties of ZnO nanostructure has been investigated [20,21,22], the elimination of cross-sensitivity of ZnO-based UV sensors to environmental humidity is still a major challenge. This is even more critical when they are made on paper substrate. As the paper is hygroscopic, the effect of moisture can cause additional significant fluctuations in the conductivity of paper-based devices. So far, the most applicable approaches to reduction of the influence of humidity include either coating paper with a superhydrophobic layer [23,24,25,26,27] or its chemical modification [28,29]. Even though those approaches are effective, they require additional processing steps, making the overall process more time-consuming and costlier. One interesting route to process paper substrate is by use of laser processing technique, which represents a simple, fast and scalable alternative to coating and chemical modification techniques and offers many advantages because of its capability of selective treatment and its fine spatial resolution [30,31,32]. It has been used to control the wetting properties of paper substrate for microfluidic applications [32,33,34]. In this work, we introduce the laser irradiation post-processing technique to withstand the influence of humidity and to improve the electrical properties of ZnO based UV detectors and screen-printed devices printed on paper substrate. Effects of the laser irradiation on the conductivity and UV sensing performance of the screen-printed ZnO nanoparticles (NPs) were studied in detail. Afterwards, both dark current and UV photocurrent of the laser treated ZnO UV sensors were measured in air with varied relative humidity (RH) to investigate the effect of water vapor on the ZnO film devices. Basic mechanisms for the observed behavior were discussed and correlated with results from structural analysis of the ZnO film. The results showed that the laser treated UV sensor had excellent compatibility between stability and sensitivity, response and recovery time, and reducing the impact of humidity.

## 2. Materials and Methods

**Figure 1:**
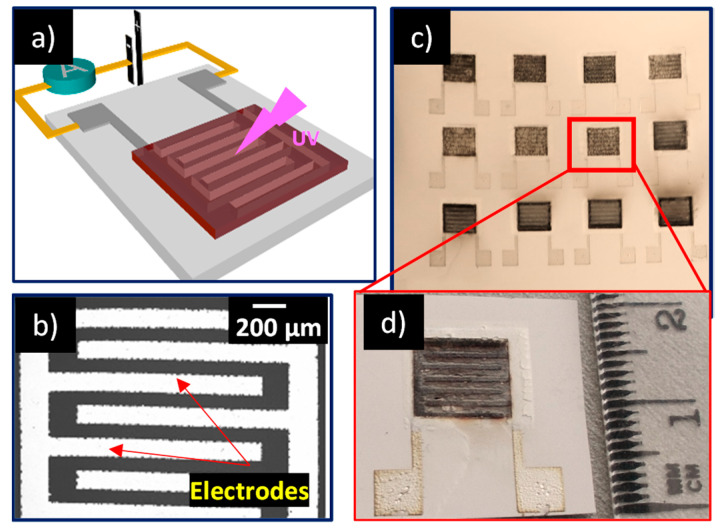
(**a**) Process sequence for fabrication of the paper-based devices; (**b**) SEM (Scanning electron microscope) image of the silver interdigitated electrodes; (**c**,**d**) Optical images of laser sintered devices fabricated on a paper substrate.

The concept of the paper-based UV sensor is based on the resistive transduction principle, consisting of a nanostructured ZnO photosensitive layer deposited on interdigitated electrodes (IDE) that are printed on paper substrate, as shown in the Figure 1a.

### 2.1. Device Fabrication

A simple, economic and scalable technological process, consisting of two screen-printing steps and a laser-postprocessing step, was used for the fabrication of the paper-based UV sensors. A schematic representation of the fabrication steps is described in the Figure S1. Firstly, a commercial silver paste (HPS-021LV, Novacentrix, Austin, TX, USA) was screen-printed with a semi-automatized screen-printer (EKRA 2H screen-printer, Dornstadt, Germany) on the paper substrate to design the IDEs and baked in an oven at 110 °C for 15 min. An individual digit of the IDE structure was 4 mm long and 170 μm wide. It was separated by a gap of 100 μm from the next digit, as shown in the Figure 1b exhibiting SEM (Scanning electron microscope, HITACHI TM3030, Tokyo, Japon) image of Ag interdigitated electrodes. 

In order to fabricate the sensitive layer, based on commercial ZnO nanoparticles (Alfa Aesar™, particle size <25 nm, Kandel, Germany), a functional paste was developed [35]. The paste composition was optimized to meet the criteria: (i) used chemicals should be cost-efficient and environmentally friendly; (ii) paste components should be suitable for laser treatment (boiling point <500 °C); and (iii) paste should produce thick uniform film on top of the electrodes, with preserved properties of ZnO nanomaterial. Main components of the paste were solvent, binder, dispersant and the nanomaterial. Water as solvent was not suitable for final application of printed structure, so ethanol was used as solvent. Since the conducted research was focused on design of sensors with minimum humidity interference, we used PVP (Poly vinyl pyrrolidone) as binder because of its slightly better stability in higher humidity conditions in comparison to other commonly used binders, such as cellulose [36]. For the dispersant, we opted to use alpha-terpineol, which is environmentally friendly, has a relatively low boiling point and is suitable for laser treatment. First, 2 g of PVP (Sigma-Aldrich, St. Louis, MO, USA) was dissolved in 10 mL of ethanol. Afterwards, 1 g of ZnO nanopowder was dispersed in 400 µL of terpineol (Sigma-Aldrich, St. Louis, MO, USA), followed by the addition of 600 µL of PVP solution. High loading ratio of nanomaterial has proven to be optimal for the screen-printing of the sensitive layer, providing desired film thickness and uniformity.

Obtained suspensions were treated with ultrasonic homogenizer (Bandelin HD-70, Berlin, Germany), operating in continuous mode at 40% of total power. Duration of ultrasonic homogenization was 10 min.

### 2.2. Laser Treatment

The laser treatment of printed films was carried out by using a diode-pumped Nd:YAG laser cutter Rofin-Sinar Power Line D–100, operating in the NIR (Near-infrared) range at 1064 nm. Frequency of the laser pulse was set at 65 kHz, and the speed of the displacement was adjusted to 500 mm s^−1^ in order to obtain sufficient pulse overlapping. The laser fluence was varied by adjusting the laser current. One sample was kept untreated to be used as a reference, and two samples were treated with the input current values of 29 A and 30 A, corresponding to laser fluences of 0.21 J cm^–2^ and 0.23 J cm^–2^, respectively. The Figure 1d shows the laser treated films sample with laser treated screen-printed ZnO nanoparticles. Figure 1c exhibits a matrix of laser treated samples, providing clear evidence that the developed process can be easily scaled-up for the large-scale fabrication of paper-based electronic devices.

### 2.3. Device Characterization

The morphology of the screen-printed ZnO film was examined by atomic force microscopy (AFM) in tapping mode. In order to precisely resolve grain boundaries, magnitude of AFM cantilever was recorded during the tapping mode imaging as well, since this signal is very sensitive to abrupt changes in the morphology. Surface roughness was calculated as a root mean square of the height distribution. The wetting characteristic of the sample was carried out by measuring contact angles using the sessile drop technique. A liquid droplet of about 2 μL in volume was dropped on the sample’s surface with a micropipette. The image of the liquid droplet was captured by a digital camera (uEye, IDS, Obersulm, Germany) attached to a microscope (Microzoom, Bausch and Lomb, Rochester, NY, USA) with 2.25× magnification and computed with the IDS camera manager software (IDS, Obersulm, Germany). The recorded droplet images were analyzed with the DropSnake Java plug-in for the ImageJ software (1.8.0, Wayne Rasband, Bethesda, MD, USA) based on B-spline snakes (active contours) to shape the drop [37]. 

## 3. Results and Discussion

### 3.1. Characterization of Laser-Treated ZnO Surface

**Figure 2:**
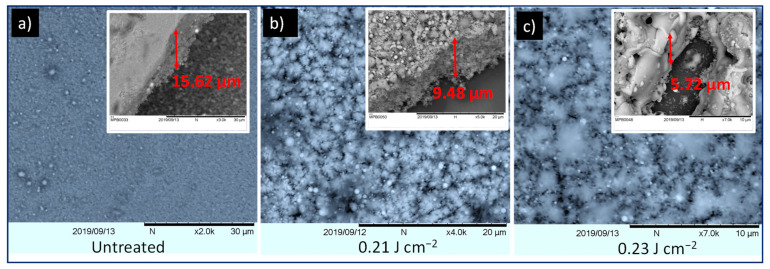
SEM images of (**a**) untreated screen-printed ZnO film, and laser-treated at (**b**) 0.21 J cm^–2^ and (**c**) 0.23 J cm^–2^. Inset: side view of untreated and treated samples.

Figure 2 exhibits top- and side-view SEM images of the ZnO film before and after laser treatment at 0.21 and 0.23 J cm^–2^ laser fluences, which clearly demonstrates that laser irradiation induces significant modifications of the film morphology. From SEM imaging of the untreated film, it can be established that nano-sized particles packed together by the dried organic additives formed a flat and uniform surface layer. When irradiated at 0.21 J cm^–2^, the film exhibited bigger pores due to the formation of melted droplets. These droplets were formed during the breaking of the large agglomerates accomplished by laser thermal evaporation of organic components. With further increase of the laser fluence at 0.23 J cm^–2^, the screen-printed film was transformed into solidified and dense ceramic material due to the complete release of the organic components and the sintering effect.

Additionally, the presented results demonstrate that the film thickness reduced from 15.6 μm to 9.5 μm and 6 μm after irradiation at 0.21, and 0.23 J cm^–2^, respectively, which is in agreement with the profile analysis provided in the supporting information (Figure S2). This reduction in film thickness can be directly correlated to the removal of organic binder components with laser radiation and the densification of the screen-printed ZnO film during the sintering process. Furthermore, it can be noticed from the EDX (Energy-dispersive X-ray) characterization, shown in Figure S3, that the Zn and O signatures are present in the untreated and laser treated samples, while carbon signature is considerably reduced in the laser treated sample. This confirms that the laser irradiation does not change the chemical composition of the ZnO nanoparticles or remove organic components of the printed paste matrix. 

**Figure 3:**
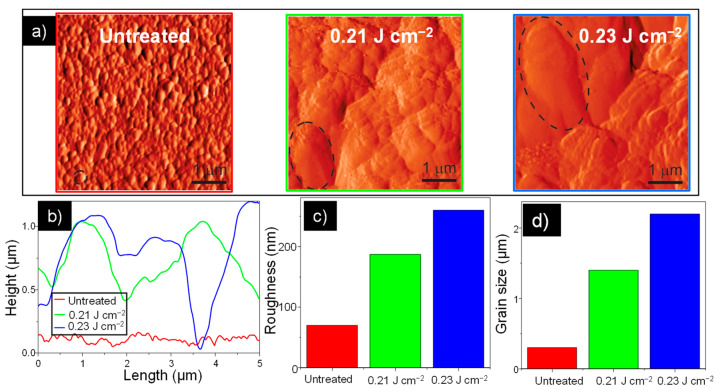
(**a**) AFM characterization of the untreated sample and samples treated at 0.21 and 0.23 J cm^–2^. Inset: Corresponding 3D morphologies. (**b**) Surface profile from AFM analysis of the untreated and laser treated samples. (**c**) Roughness measurement of the investigated screen-printed films. (**d**) Grain size measurement of the investigated screen-printed films.

Therefore, additional investigations were performed by atomic force microscopy (AFM) in tapping mode to explore the change of surface morphology in the laser sintered samples. Figure 3a presents images of the AFM magnitude of the untreated and all treated samples, which emphasize grains and grain boundaries. As can be seen, the morphology significantly changes from a flat surface with small grains in the case of the untreated sample, to very rough surfaces with large grains in the case of the treated samples. Therefore, these images clearly represent the sintering process where small grains are merged into bigger ones. The underlying morphological changes during the sintering are summarized in the bottom row of Figure 3, in which Figure 3b shows characteristic height profiles from topographic images, while Figure 3c displays the surface roughness. An average grain diameter of the selected grains (encircled by dashed lines in the magnitude images) is presented partly in Figure 3d. The height profile of the untreated sample was a rather flat line, the surface roughness was around 30 nm, while the diameter of the selected grain was around 270 nm. On the other hand, the surface roughness of the treated samples were in the range of 200–300 nm, with some grains being larger than 2 µm. 

**Figure 4:**
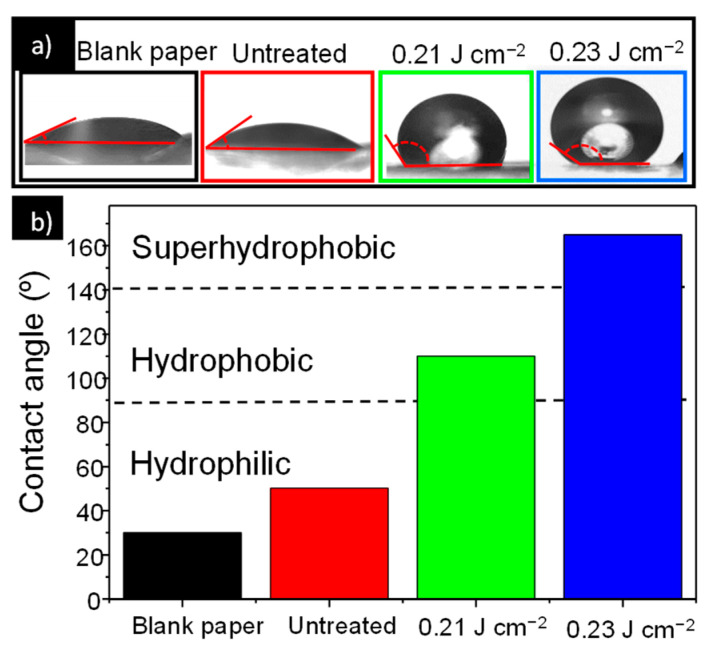
(**a**) Photographs of water droplet shape on the untreated and laser treated ZnO screen printed films. (**b**) Contact angle measured for untreated and laser treated samples.

Figure 4 shows the contact angle of the untreated sample and samples treated with 0.21 and 0.23 J cm^–2^ laser fluence, where it can be noticed that hydrophobicity of the ZnO film increases with increase in the laser fluence. Clearly, a water droplet stands stable on the laser treated films, while it spreads out on the untreated sample. The wettability behavior of a surface is strongly related to the surface morphology [38]. From the AFM measurements, we established that morphology and roughness of the film surfaces change with the increase of the laser fluence. The surface of the untreated film (Figure 4a) with low surface roughness shows a low contact angle with hydrophilic behavior. With the rise of the surface roughness the contact angle increased, indicating a hydrophobic behavior for the sample treated at 0.21 J cm^–2^ and super hydrophobic behavior for the sample treated with higher laser fluence 0.23 J cm^–2^. For porous nanostructured surfaces, the Cassie and Baxter (CB) model is usually applied for the description of wetting properties [39]. According to the CB model, the water contact angle on the porous surface is greatly influenced by the surface fraction of solid–liquid and liquid–air interfaces on the solid surface. In the CB wetting state, the large volume of air trapped between the grains prevents the water droplet from penetrating into the free space and the water droplet is suspended above the substrate unstably. Within such a framework, a hydrophilic surface can be modified into a hydrophobic one, and the contact angle increases with the ratio of water–air interface to the total area because air pockets formed below the water droplet minimize the interfacial energy. Considering that surface roughness of ZnO films increase with laser treatment, the fraction of interfacial area between air and liquid also increases. For highly rough surfaces, more air can be trapped within the interstices, increasing measured contact angle. The increased surface roughness and additional trapped air prevent further penetration of the water droplet into the solid surface, giving rise to the observed shift from hydrophilicity to super-hydrophobicity.

### 3.2. UV Detection Performances

**Figure 5:**
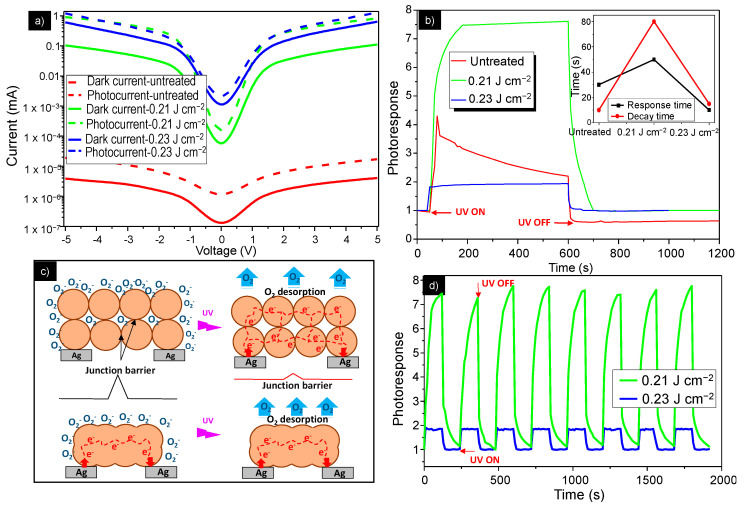
(**a**) I–V curves for uncoated and untreated samples and samples sintered at 0.21, and 0.23 J cm^–2^ with and without UV illumination. (**b**). Dynamic response behavior (response/recovery times) for the untreated and laser treated samples under UV illumination (**c**) Schematic diagram of the NP–NP junctions. (**d**) Time-resolved photoresponse of the ZnO device treated at 0.21 and 0.23 J cm^−2^ under a continuous UV light rectangle pulse.

Figure 5a displays the dark current–voltage (I–V) characteristics, together with the photocurrent of the untreated samples and samples sintered with 0.21 J cm^−2^ and 0.23 J cm^−2^ laser fluences, measured in the −5 V to 5 V range at ambient humidity of 45%. Analysis of the obtained results clearly reveals that increase in laser fluence induces a significant increase in the dark current values, thus improving the conductivity of the printed ZnO film. Indeed, for the untreated sample, the profile of the I(V) curve corresponds to that of the material with poor conductivity with 3.42 nA at 5 V, whereas the I(V) curve of the sample treated with 0.23 J cm^–2^ has 5 orders of magnitude higher current (935 μA at 5 V). For porous nanomaterials, where nanoparticles are packed together, the conductivity is greatly affected by a mechanism governed by the grain-boundary resistance, since the resistance at these contacts is much higher than the resistance across the grains. The conduction channels in the ZnO–NP film include NP–NP junctions, and electrons must overcome the junction barriers to pass from one nanoparticle to another. Using AFM measurements, it was determined that during the sintering process, most of these grain boundaries vanished as the nanoparticles form neck like structures and the grains connected, reducing the junction barrier and creating a conductive path for electron transfer. In this case, the grain conductivity becomes dominant, leading to an observed increase in the current value. 

Figure 5a also demonstrated that there was a significant increase in the photocurrent of all the samples when they were exposed to 360 nm wavelength at 10 mW cm^–2^. This excitation wavelength was chosen to be in the vicinity of the optical band gap of ZnO (Eg = 305–395 nm) [40]. Moreover, it was noticed that a relatively high photocurrent (≈300 nA) was obtained at zero bias for the sample treated at 0.23 J cm^−2^, offering the possibility to use this laser treated device as a self-powered, paper-based photodetector system. The Figure 5b depicts the photocurrent-to-dark current ratio (called the photoresponse) as a function of the time, which compares the switching characteristics of the investigated samples under a bias of 5 V. Figure 5b demonstrated that a significant increase in the photoresponse was observed under UV light illumination for the untreated samples, as well as for the samples treated at 0.21 and 0.23 J cm^−2^, confirming the results from Figure 5a. 

However, the untreated device showed poor stability, with photoresponse decreasing gradually after the light source was switched on, while the laser treated samples showed good stability with constant photocurrent values under UV illumination, and a return to the initial value after switching off the light source. This can be attributed to some residual organic additives in the untreated screen-printed film, resulting in a poor conducting path for the electron transfer between nanoparticles, thus generating unstable conductivity. During the sintering process, the organic additives were removed from the film, creating better ZnO nanoparticle interconnection and thus the conducting path for electron transfer.

The device sintered at 0.21 J cm^−2^ shows better photosensitivity than the film sintered at 0.23 J cm^–2^, which is due to its more efficient structure and morphology. The processes of generating and transporting carriers in the laser sintered devices are illustrated in the schematic diagram shown in Figure 5c. The photodetection property of the ZnO NPs-based screen-printed film is strongly influenced by two mechanisms: the adsorption and desorption of oxygen in the air. In the absence of photons, oxygen molecules adsorb on the surface of the ZnO nanoparticles as negatively charged ions, by capturing free electrons from the n-type ZnO. This process creates a low-conductivity depletion layer near the surface of the NPs. When the film was irradiated with UV light, electron-hole pairs were created in the depletion region and the negatively charged surface species traps the holes and releases the electrons into the conduction band of ZnO, so the current gradually increases until saturation. The conduction channels in the ZnO NPs detectors also include NP–NP junctions. Electrons must get over the junction barrier to pass from one NP to another. These barriers formed by the surface depletion layers can govern the charge transport within the film under UV illumination. 

When treated at 0.23 J cm^–2^, the film becomes more compact and bulkier, with bigger grains, lower porosity and thickness reducing surface-to-volume ratio of the film structure compared with the structure of the film treated at 0.21 J cm^–2^. This facilitates the penetration of oxygen species into the inner film layers, ensuring the participation of the nanoparticles from whole film in the UV sensing mechanism. Therefore, under illumination, the film treated with 0.23 J cm^–2^ fluence provides less active surface area and lower rate of oxygen desorption, resulting in a lower photocurrent. Additionally, the structure of the NP–NP junction is beneficial for the electrons to flow through the nanoparticle networks under UV light illumination, giving rise to the increase in photocurrent. 

The inset in Figure 5b shows decay and rise time of the untreated and laser treated devices, defined respectively as the time required for photocurrent to drop from 90% to 10% and rise from 10% to 90% of its maximum value. The sample treated at 0.23 J cm^–2^ had a shorter response time than the sample treated at 0.21 J cm^−2^, with decay and rise time of 10 s, which is comparable with other paper-based UV-detectors [8,41].

Furthermore, in order to examine the repeatability of the screen-printed paper-based ZnO UV detectors, the time-resolved photoresponse at 5 V bias with multiple UV on/off cycles was measured, in which both the turn-on and turn-off times of the UV light equaled 2 min. The Figure 5d shows height cycles of photocurrent switching, demonstrating very good repeatability and sensitivity of the two laser treated photodetectors.

### 3.3. Suppressed Response to Humidity by Laser-Post Processing Treatment

**Figure 6:**
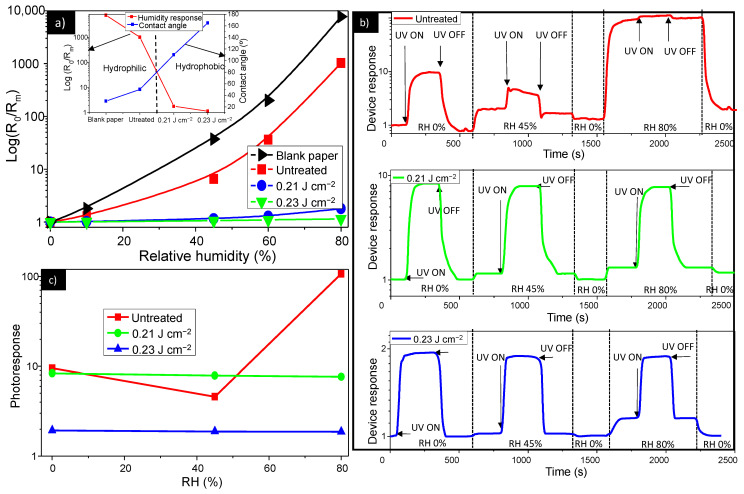
(**a**) Electrical resistance changes for the untreated and laser treated devices as a function of RH (Relative Humidity). Inset: Humidity response at 60% and contact angle as a function of the laser fluence. (**b**) Dynamic response of untreated and treated ZnO sensor to UV light at various humidity levels. (**c**) Photoresponse of the untreated and laser treated samples as a function of RH.

The influence of humidity was evaluated by calculating the device’s response given by the ratio of the initial electrical resistance (R_0_) at zero humidity, used as a baseline, to the electrical resistance when humidity is introduced (R_m_), measured between the IDE. The inclusion of an uncoated paper allowed us to assess whether the ZnO screen printed film coating suppresses the water vapor absorption. The results shown in Figure 6a disclose the strong response differences between different samples, clearly indicating the effects of the screen-printed films and laser surface modification. For the uncoated paper, an important rise of the response is observed when humidity increases, with two times increased response with increasing RH from 0 to 10%, and at 3 orders of magnitude higher at moderate humidity levels (60%) than at 0% RH. The untreated ZnO sample shows a lower sensitivity to humidity than the uncoated sample. However, the sensitivity to humidity of the untreated sample was still significant, at 2 orders of magnitude higher at moderate humidity levels (60%) than at 0% RH. It was evident that the samples treated with laser exhibit a negligible response to humidity, revealing a very poor sensitivity to humidity of the laser treated ZnO films. This difference in humidity sensitivity between the untreated and laser treated samples can be directly correlated to their different surface wettability properties. The inset in Figure 6a depicts the relationship of the contact angle and response to humidity in the investigated samples. It was noticed that the samples with hydrophobic surfaces were sensitive to humidity, while humidity did not affect the laser treated samples with hydrophilic surfaces. Indeed, when the sensor film was hydrophilic, the water nucleation barrier was low, yielding high nucleation rate due to the strong attraction forces between the surface and the water molecules. This implies that water vapor is strongly adsorbed onto the sample surface, resulting in high humidity response. When the sensor film was hydrophobic, the energy barrier was high, thus minimizing the water vapor adsorption onto the film surface, which resulted in roughness induced non-wetting properties and no humidity interference. 

Figure 6b showed the UV photoresponse of the investigated samples cross-linked with different humidity levels of 0%, 45% and 80%. It was noticed that the photoresponse of the untreated samples were greatly affected by the presence of water molecules, whereas the effect of the humidity on the photoresponse of the laser treated samples can be neglected. Indeed, the photoresponse of the untreated sample decreased with the humidity increase. This was due to the fact that water molecules replaced the previously surface adsorbed ionized oxygen species, and hence released electrons into the conduction band, a process which partially annihilates the depletion layer, leading to a rise in the dark current, as shown in the Figure 6a. Under the UV illumination, the dissociated H_2_O molecules on the surface of the ZnO film capture both electrons and holes generated by UV light, leading to a decrease of carrier density and thus lower photoconductivity. At low RH <50%, the effect of O_2_ desorption is more pronounced, so that the photoresponse slightly decreased with the humidity but it was still detectable. At high RH, around 80%, the effect of the dissociated H_2_O capturing electrons and holes becomes more significant due to a discrete water layer formed on the ZnO film surface, which generates a humidity-induced degradation of ZnO-based photodetector with a photoresponse almost undetectable. As seen in Figure 6c, in the case of the laser treated samples, the photoresponse does not change with the humidity level, due to the roughness engineered hydrophobic surface that hampers water molecules to be absorbed at the surface of the ZnO film, as previously explained.

The laser-profiling of the sensitive layer surface roughness provides us with a powerful tool for the management of the film wettability properties, with high impact on tuning of the device sensitivity toward humidity. The undertaken approach can be easily optimized for fabrication of humidity-sensitive ZnO film that can be used for humidity sensors or for fabrication of paper-based ZnO film with no humidity interference that can be used for other electronic devices like UV detectors.

## 4. Conclusions

In summary, we developed a new strategy to reduce humidity interference on paper-based UV sensor. The proposed approach is fast, cost-effective, scalable, easy-to-operate and paper-friendly, since they do not require annealing steps at high temperatures. The effects of the laser fluence on morphology, electrical properties, UV and humidity sensing properties were disclosed. The ZnO film conductivity and UV photoresponse were improved and the influence of the humidity was considerably reduced by properly adjusting the laser fluence. It was found that this phenomenon is correlated to the change of the morphology and structure of the ZnO film generated by the laser irradiation. Additionally, the resulting UV sensors showed good repeatability and relatively short response time. Therefore, the possibility of fabricating paper-based sensing devices with no humidity impact in a rapid and large-scale manner paves the way to low-cost solutions of sophisticated paper-based electronics with low environmental footprint.

## Data Availability

The data presented in this study are available on request from the corresponding author.

## Supplementary Materials

The following are available online at https://www.mdpi.com/2079-4991/11/1/80/s1, Figure S1: Process sequence of the humidity sensors, Figure S2: Thickness profile of the ZnO films, Figure S3: EDX profiles of the untreated and treated ZnO films.
