# Supplementary material for: Laser-Tunable Printed ZnO Nanoparticles for Paper-Based UV Sensors with Reduced Humidity Interference
Source: Nanomaterials (Basel). 2021 Jan 2;11(1):80. doi: 10.3390/nano11010080 (PMC7824355; doi:10.3390/nano11010080)
Supplement: Supplementary file 1 [file nanomaterials-11-00080-s001.pdf]

Supplementary Materials

Laser-Tunable Printed ZnO Nanoparticles for Paper-Based UV Sensors with Reduced Humidity Interference

[1] Center for Sensor Technologies, BioSense Institute, University of Novi Sad, Zorana Đinđića, 21101 Novi Sad, Serbia; marrad@biosense.rs
[2] Institute of Physics Belgrade, University of Belgrade, Pregrevica 118, 11080 Belgrade, Serbia; bvasic@ipb.ac.rs
* Correspondence: georges.dubourg@biosense.rs

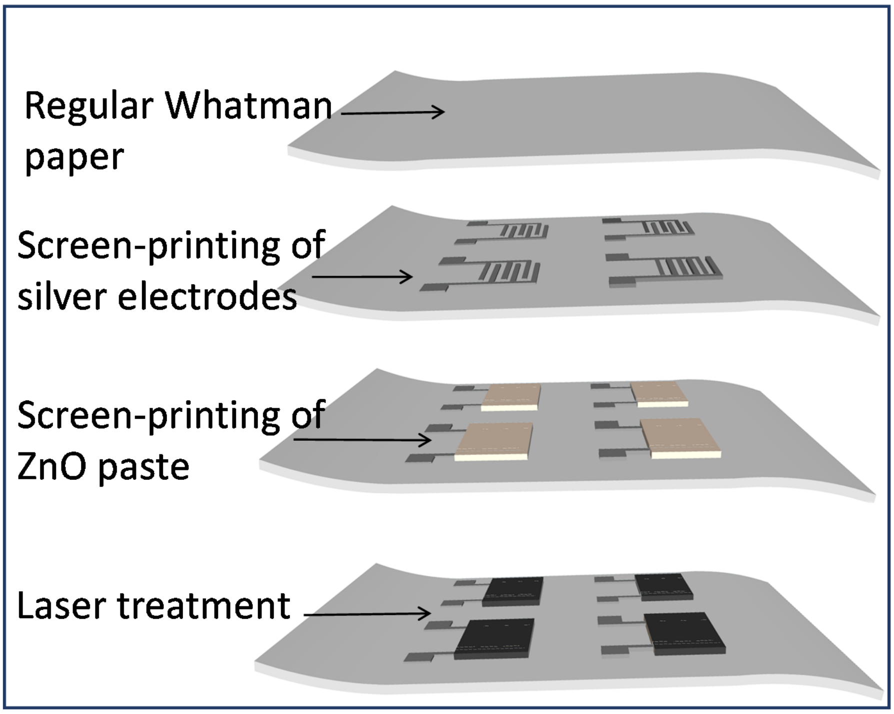

Figure S1. Process sequence of the ZnO-based UV sensor printed on paper substrate.

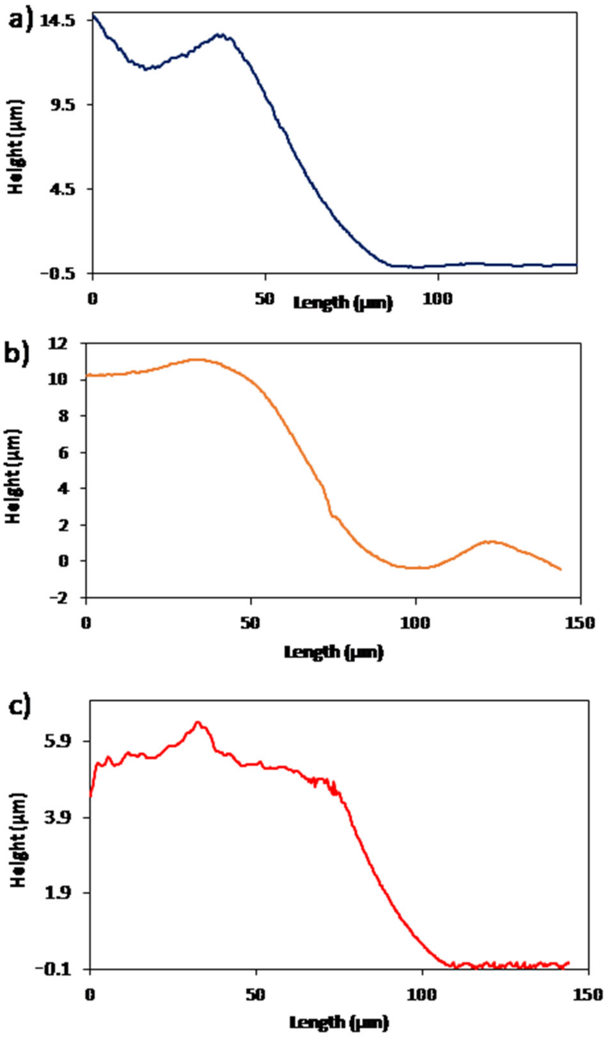

Figure S2. Thickness profile obtained by three-dimensional (3D) image Viewer (HITACHI) of (**a**) untreated film, and film treated at (**b**) 0.21 and (**c**) 0.23 J cm^{-2} laser fluence.

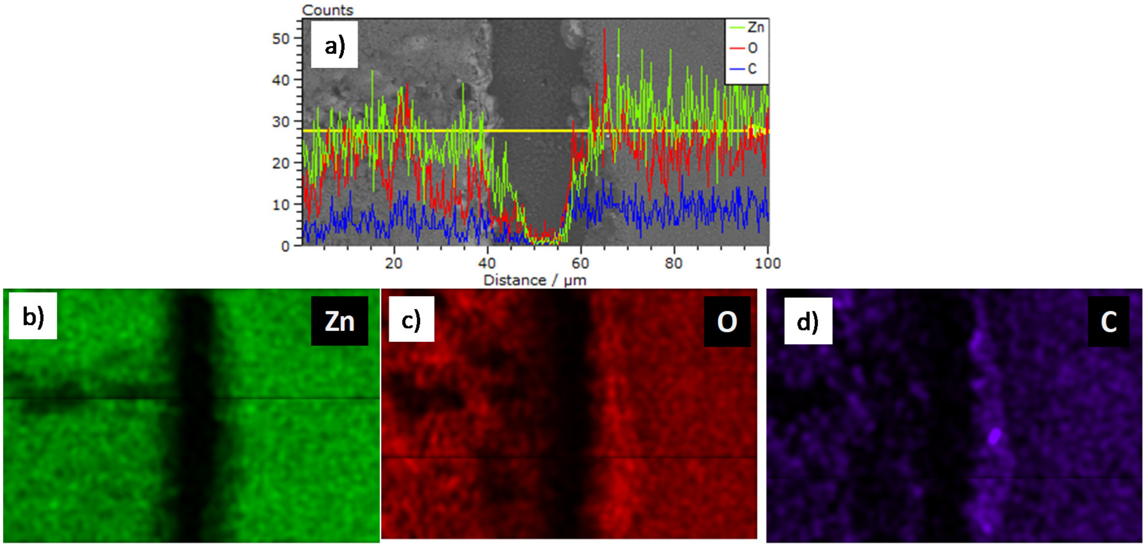

Figure S3. (**a**) SEM image of the laser sintered 0.23 J cm^{-2} (left) and untreated surface (right) of screen-printed film with corresponding zinc, oxygen and carbon EDX profiles. (**b–d**) Corresponding zinc, oxygen and carbon EDX maps of untreated and treated parts.
